# Supplementary figures and images for: Towards a comprehensive view of the pocketome universe—biological implications and algorithmic challenges
Source: PLoS Comput Biol. 2025 Jul 24;21(7):e1013298. doi: 10.1371/journal.pcbi.1013298 (PMC12324681; doi:10.1371/journal.pcbi.1013298)

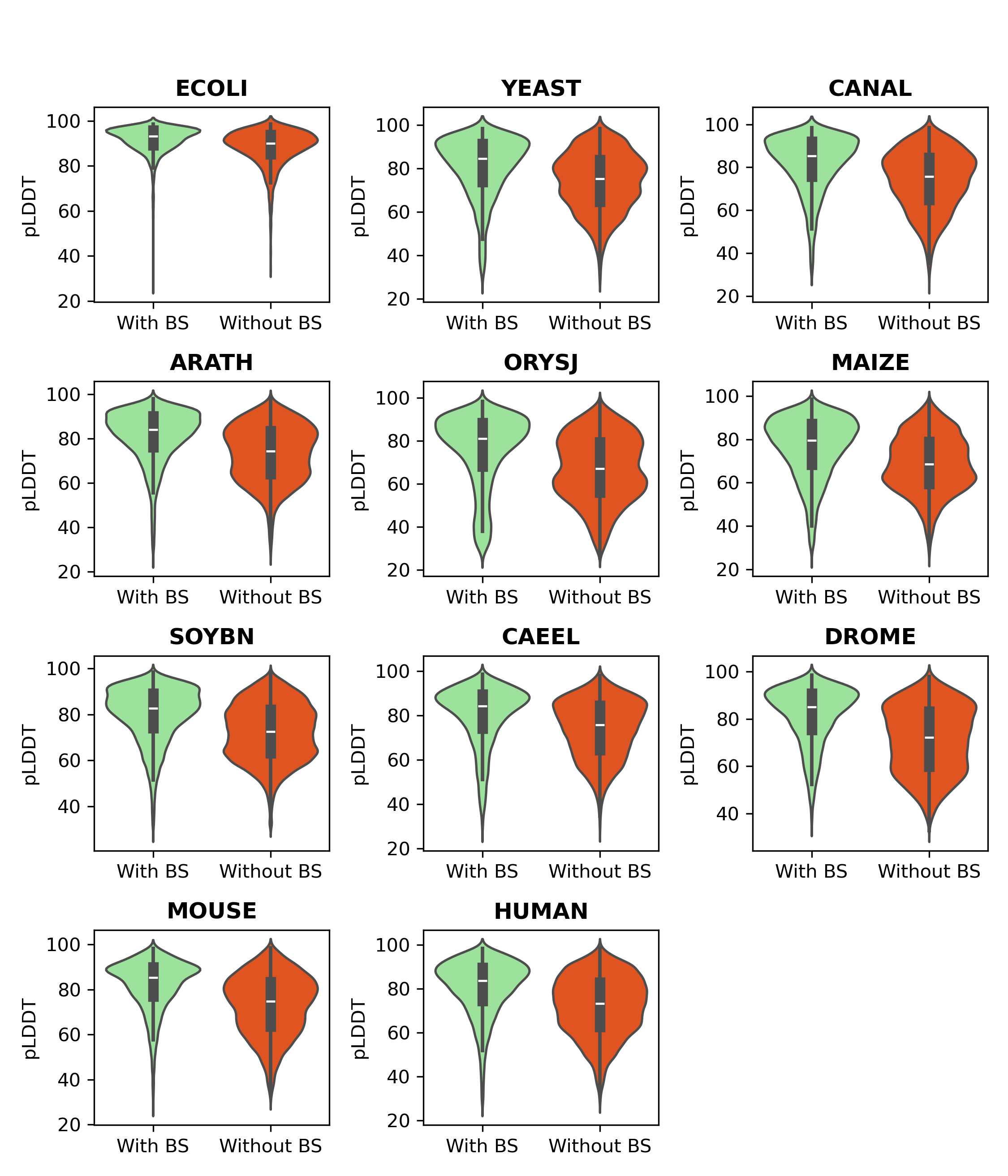

Supplement: S1 Fig — The highest median pLDDT for proteins with and without binding sites was observed for ECOLI, with 93.24 and 90.07, respectively. The lowest median pLDDT of proteins with binding sites was observed for MAIZE with 79.41 and for ORYSJ of proteins without binding sites with 67.05. The median pLDDT values of proteins for the other species range from 80.95 to 85.40 for proteins with binding site and from 68.48 to 75.75 for proteins without any binding site predicted by P2Rank. (TIFF) [file pcbi.1013298.s001.tiff]

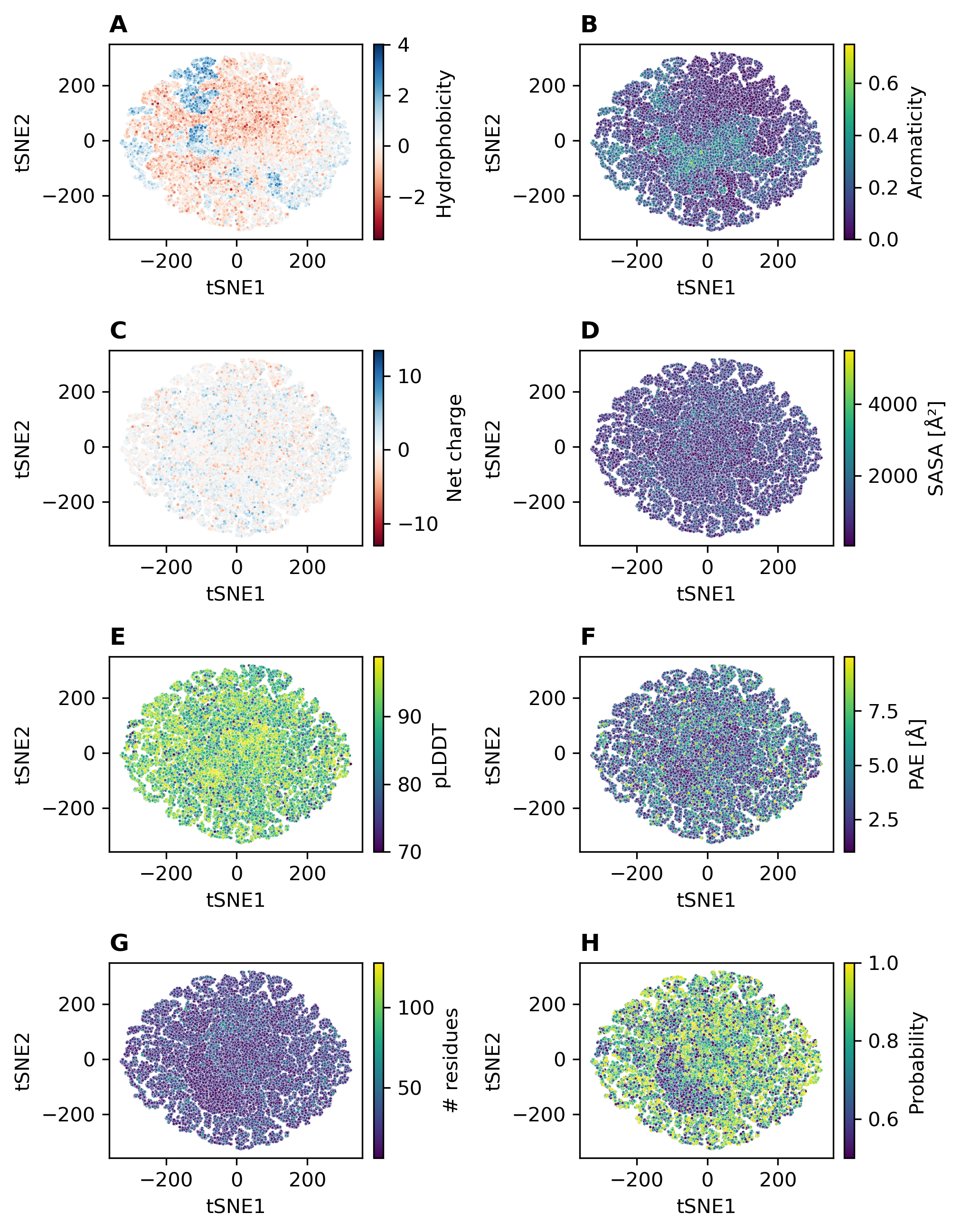

Supplement: S2 Fig — (TIFF) [file pcbi.1013298.s002.tiff]

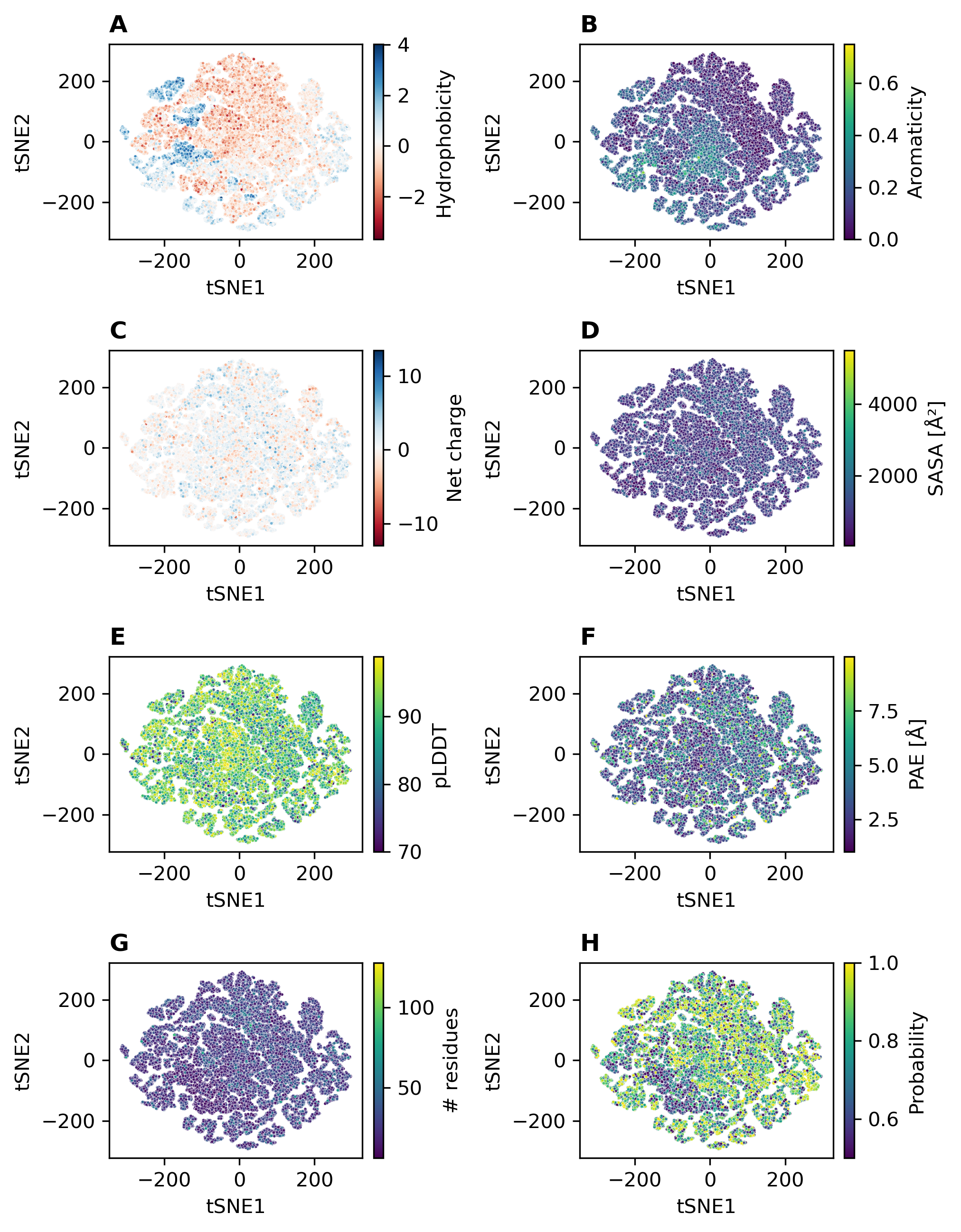

Supplement: S3 Fig — (TIFF) [file pcbi.1013298.s003.tiff]

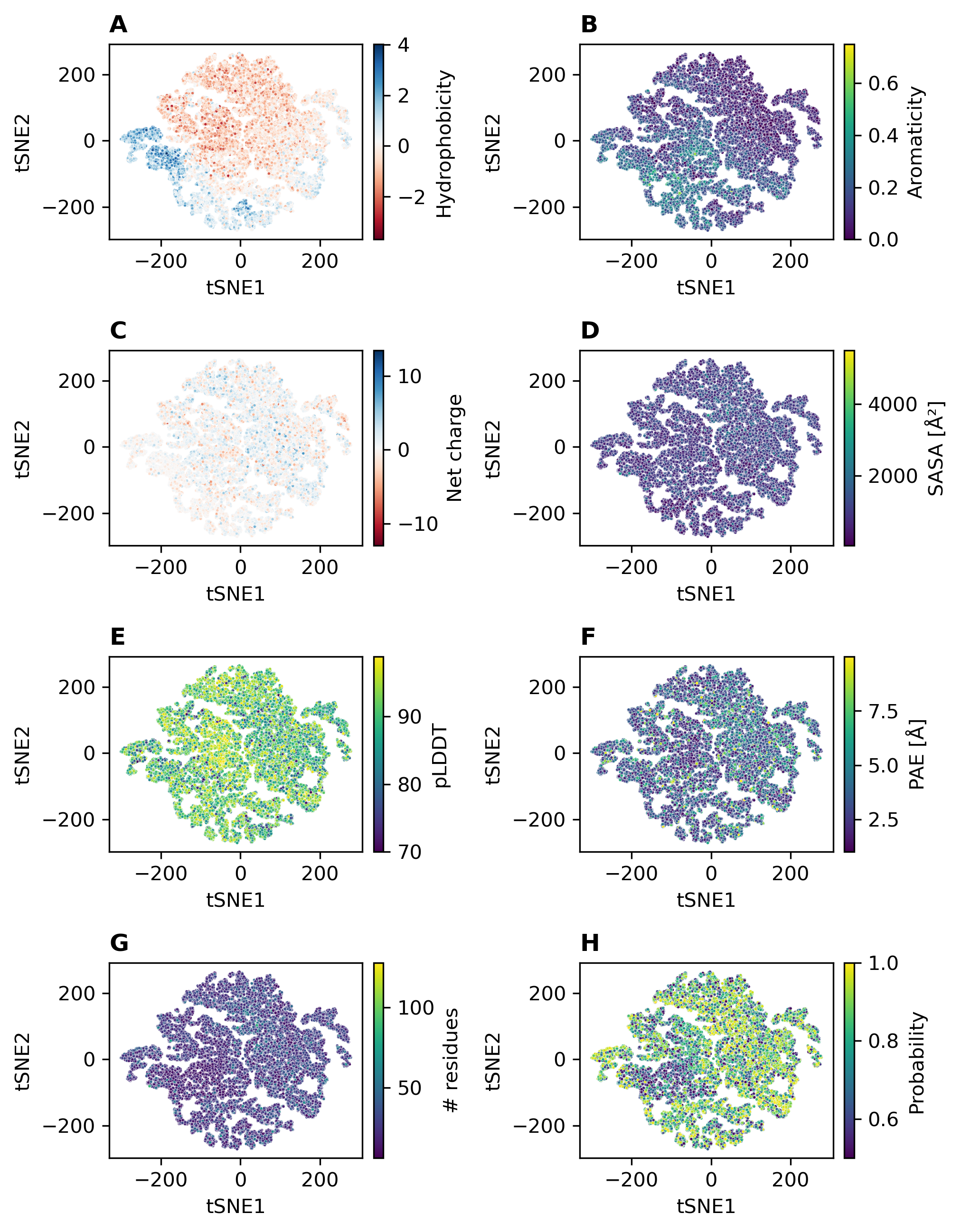

Supplement: S4 Fig — (TIFF) [file pcbi.1013298.s004.tiff]

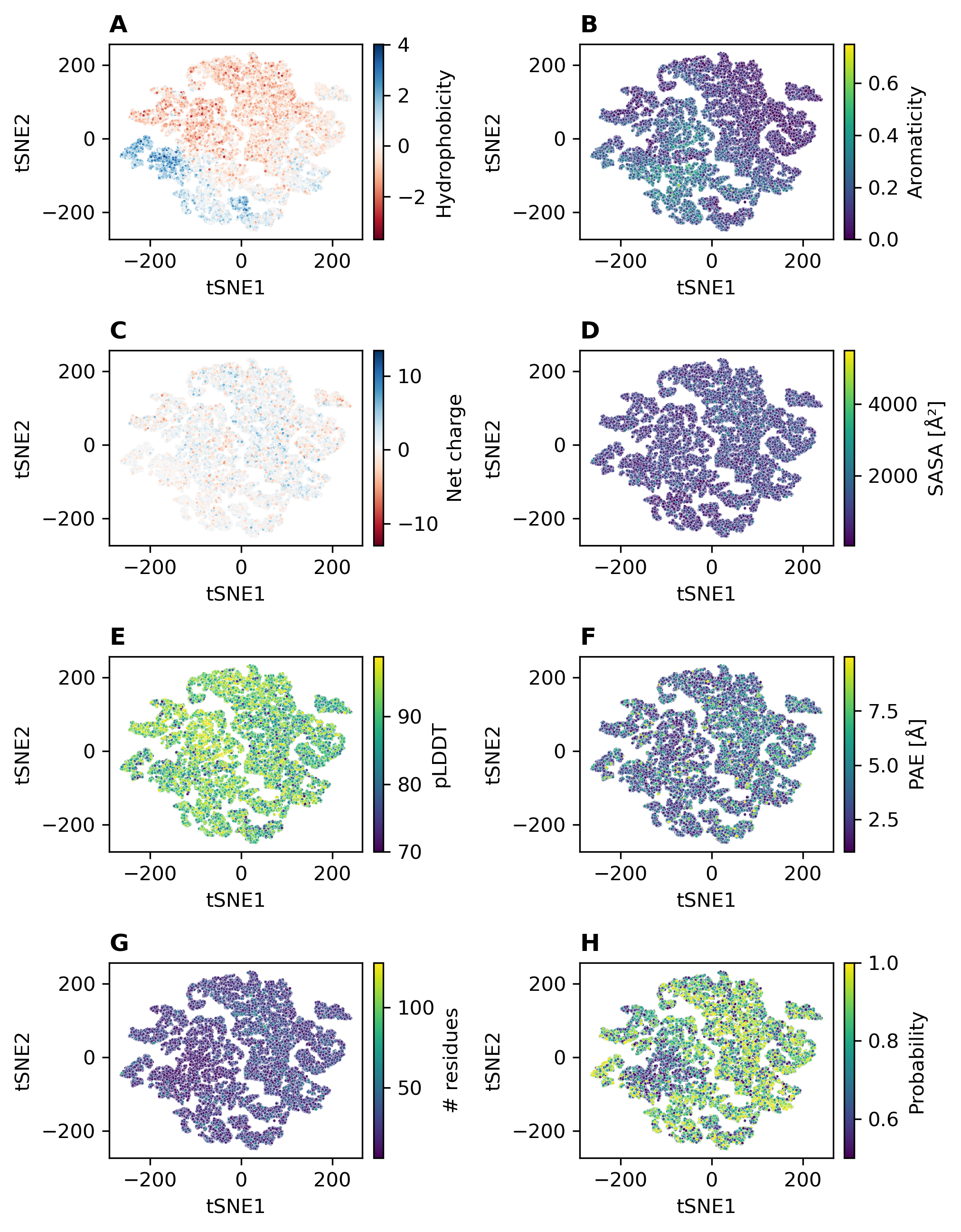

Supplement: S5 Fig — (TIFF) [file pcbi.1013298.s005.tiff]

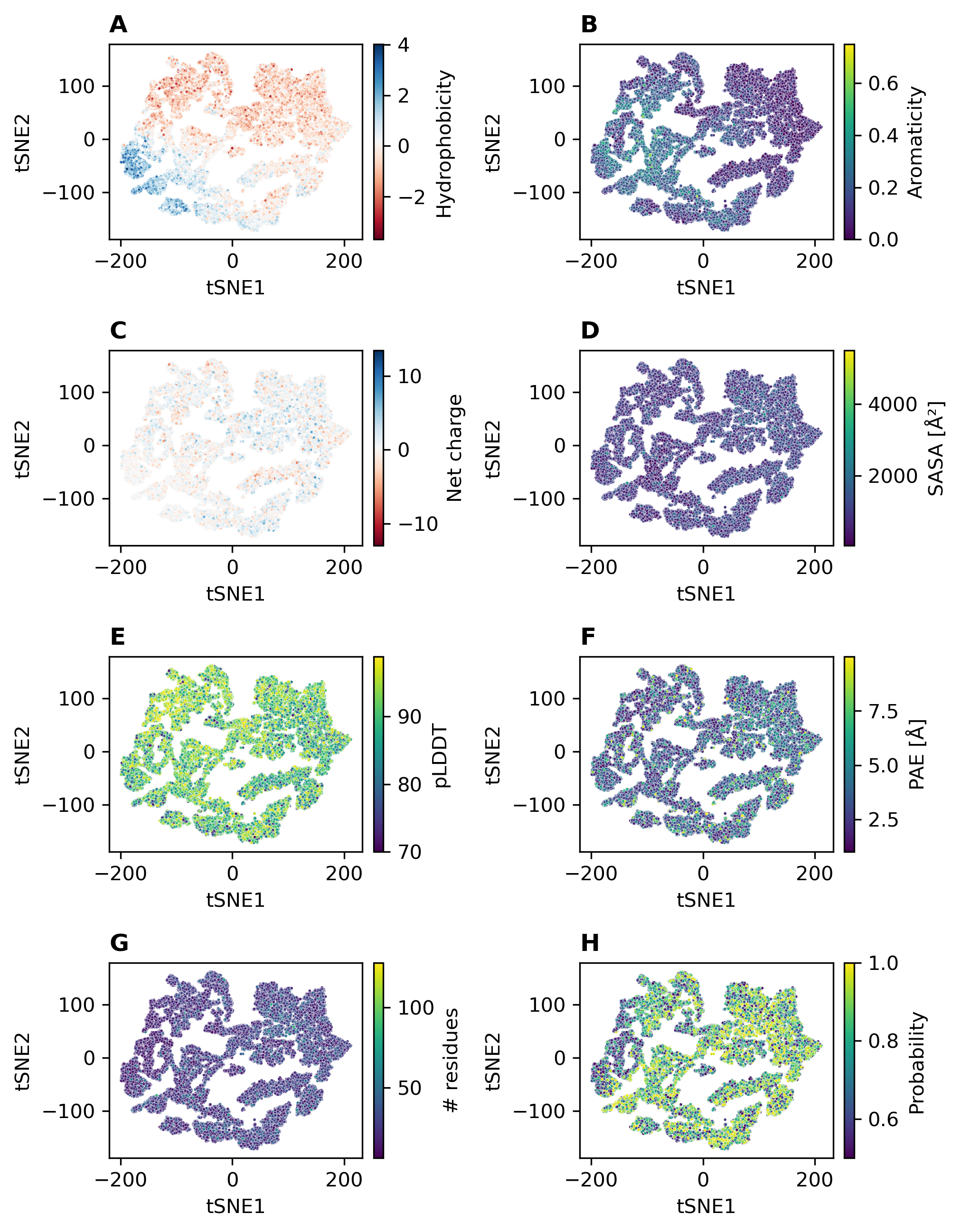

Supplement: S6 Fig — (TIFF) [file pcbi.1013298.s006.tiff]

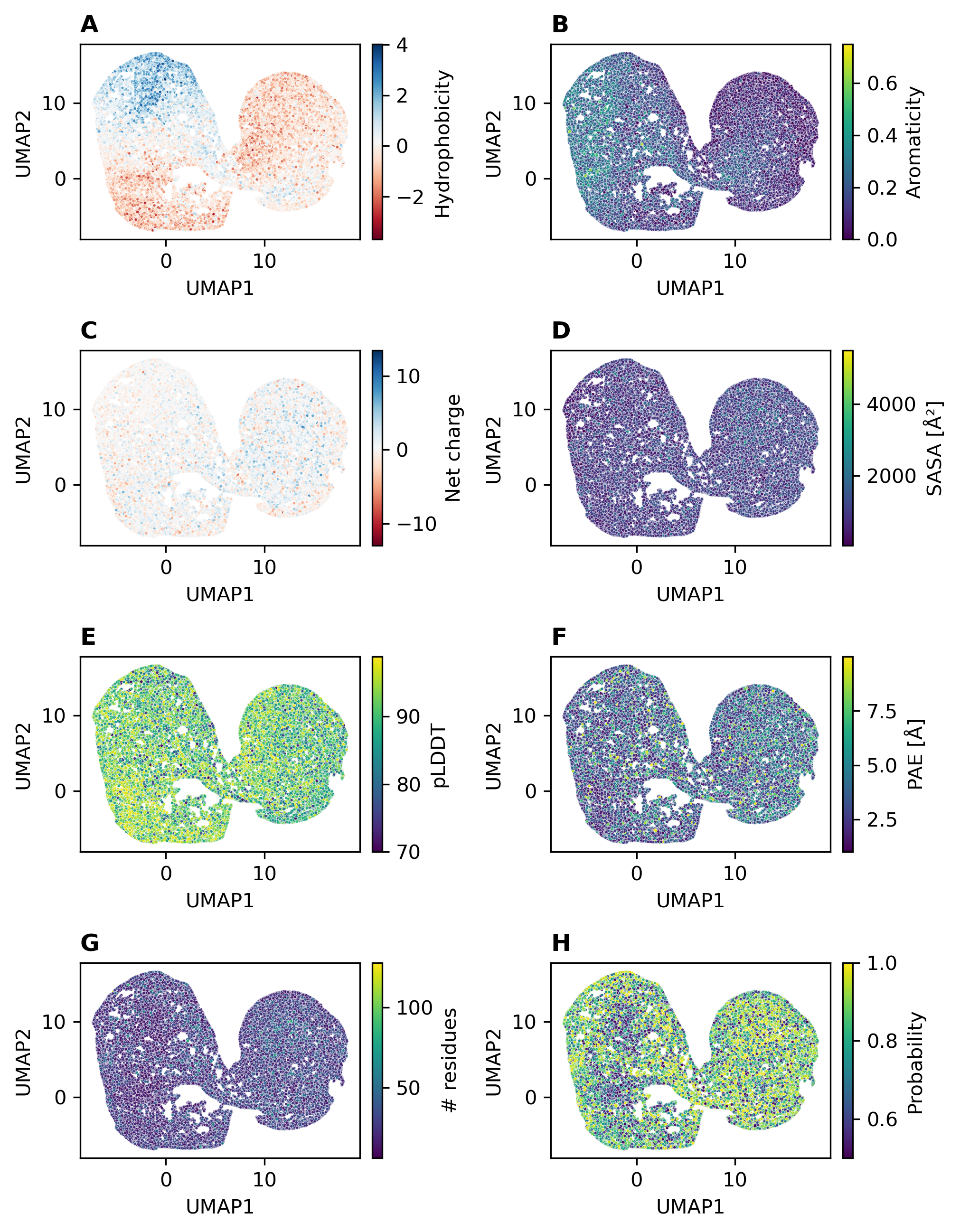

Supplement: S7 Fig — UMAP plots (n_neighbors = 10) for all predicted pockets of all 11 species colored by different properties. (TIFF) [file pcbi.1013298.s007.tiff]

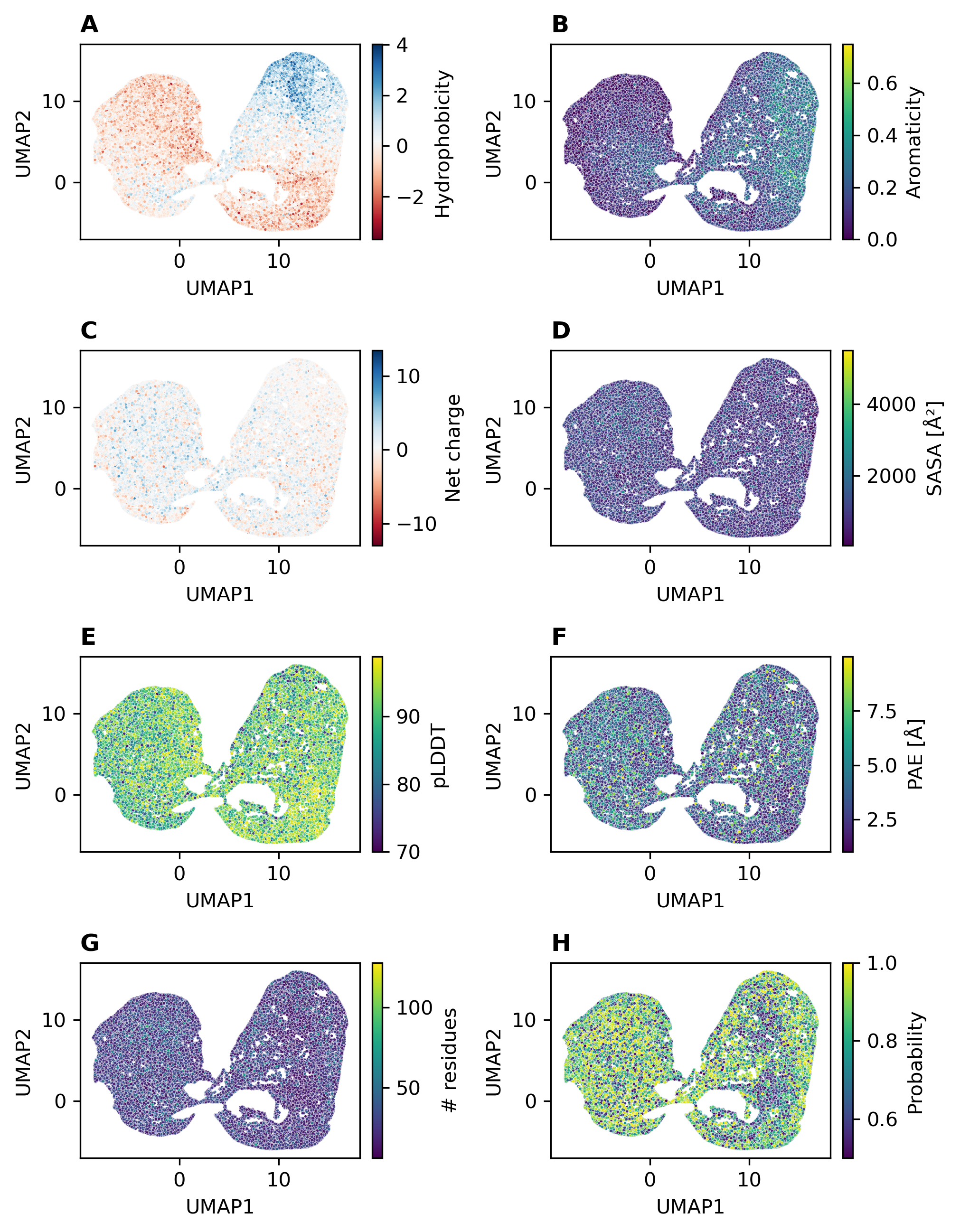

Supplement: S8 Fig — UMAP plots (n_neighbors = 20) for all predicted pockets of all 11 species colored by different properties. (TIFF) [file pcbi.1013298.s008.tiff]

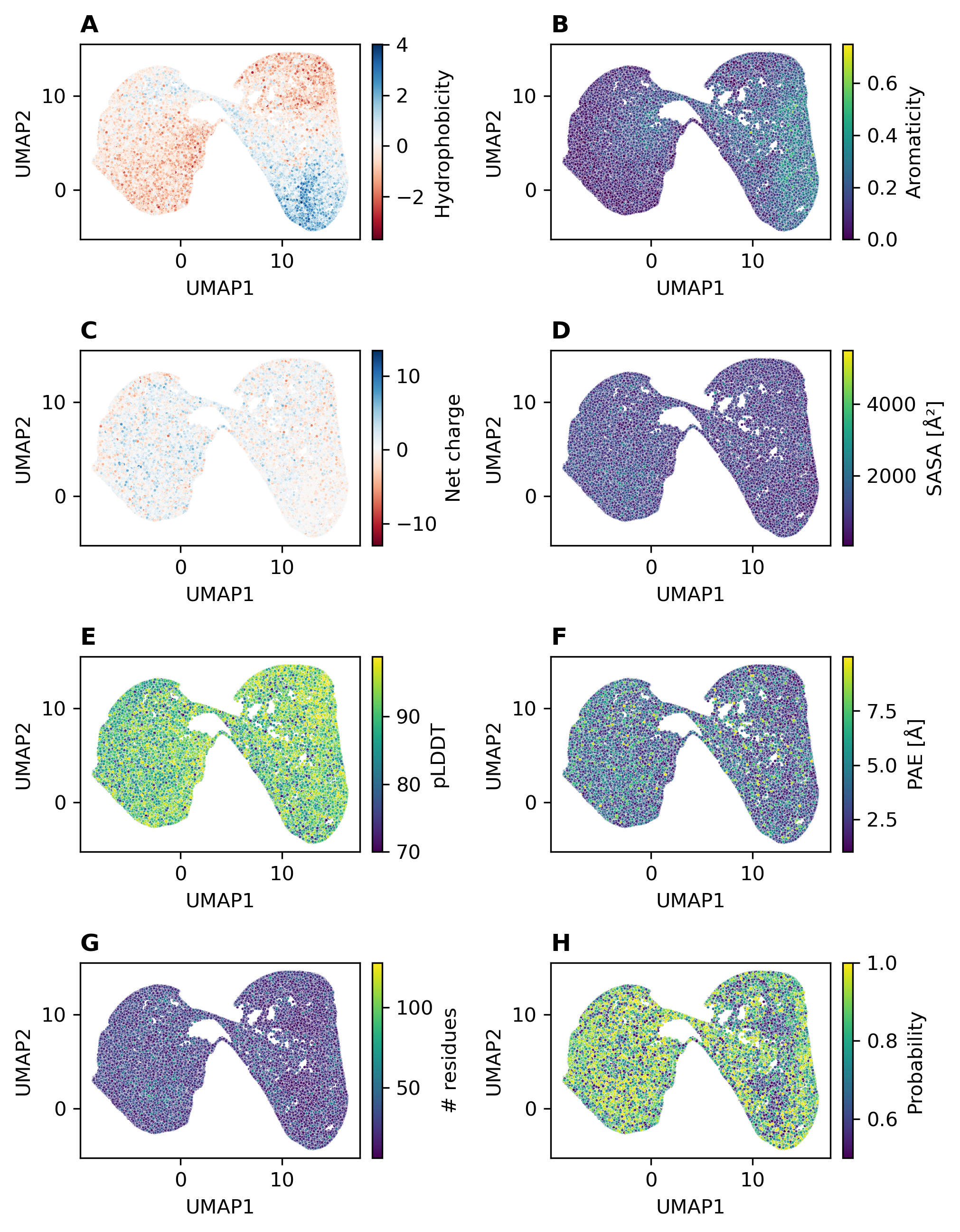

Supplement: S9 Fig — UMAP plots (n_neighbors = 50) for all predicted pockets of all 11 species colored by different properties. (TIFF) [file pcbi.1013298.s009.tiff]

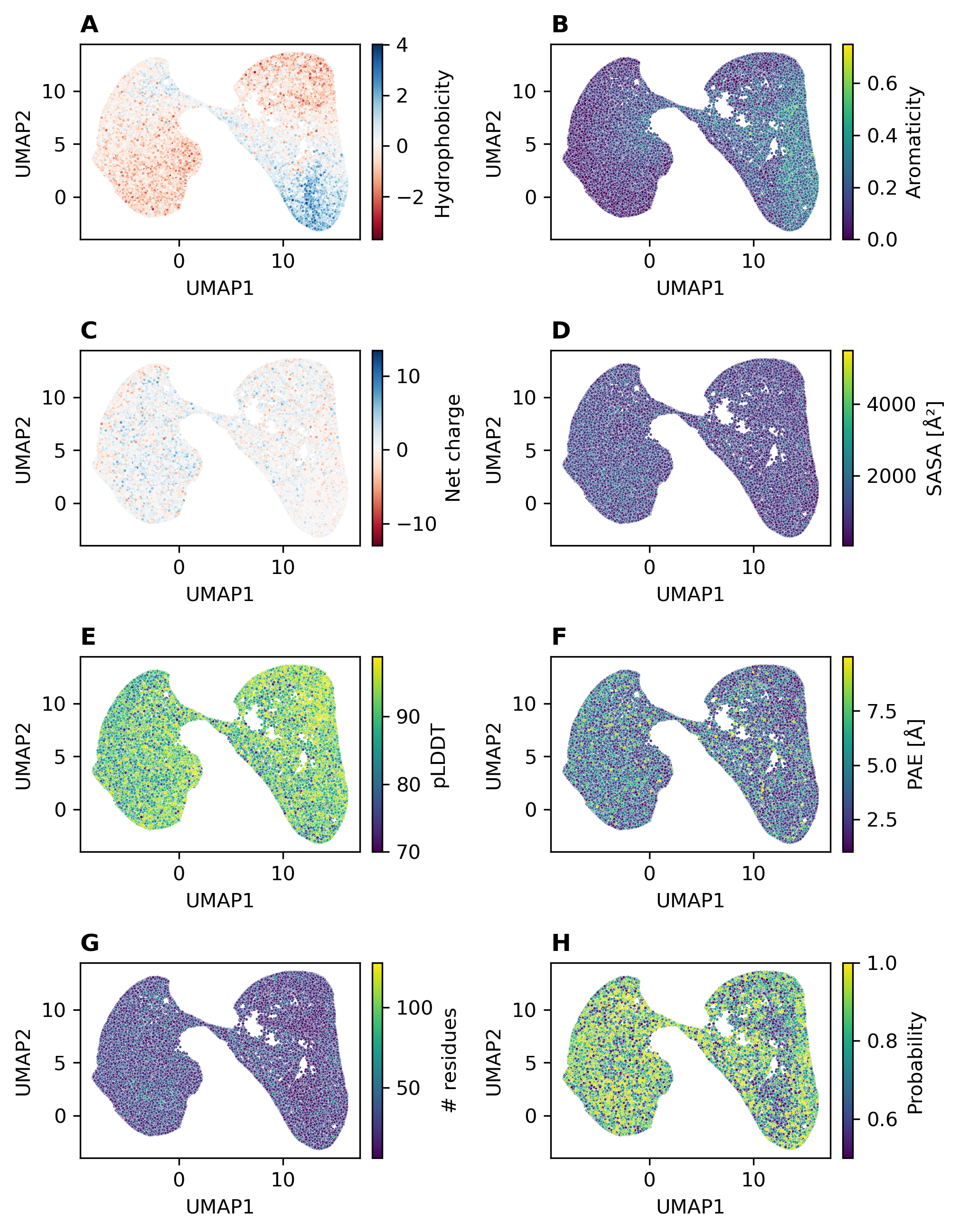

Supplement: S10 Fig — UMAP plots (n_neighbors = 100) for all predicted pockets of all 11 species colored by different properties. (TIFF) [file pcbi.1013298.s010.tiff]

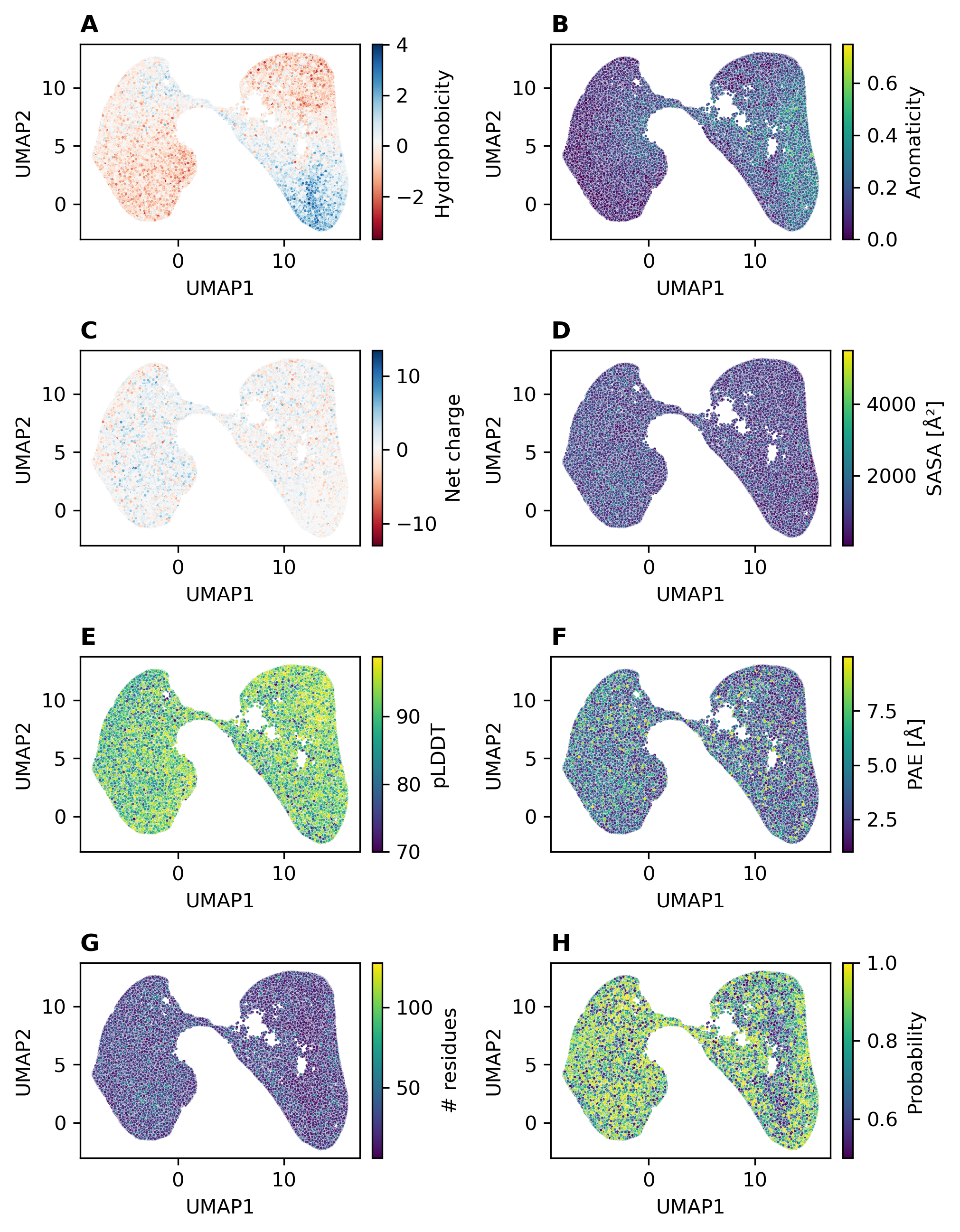

Supplement: S11 Fig — UMAP plots (n_neighbors = 200) for all predicted pockets of all 11 species colored by different properties. (TIFF) [file pcbi.1013298.s011.tiff]

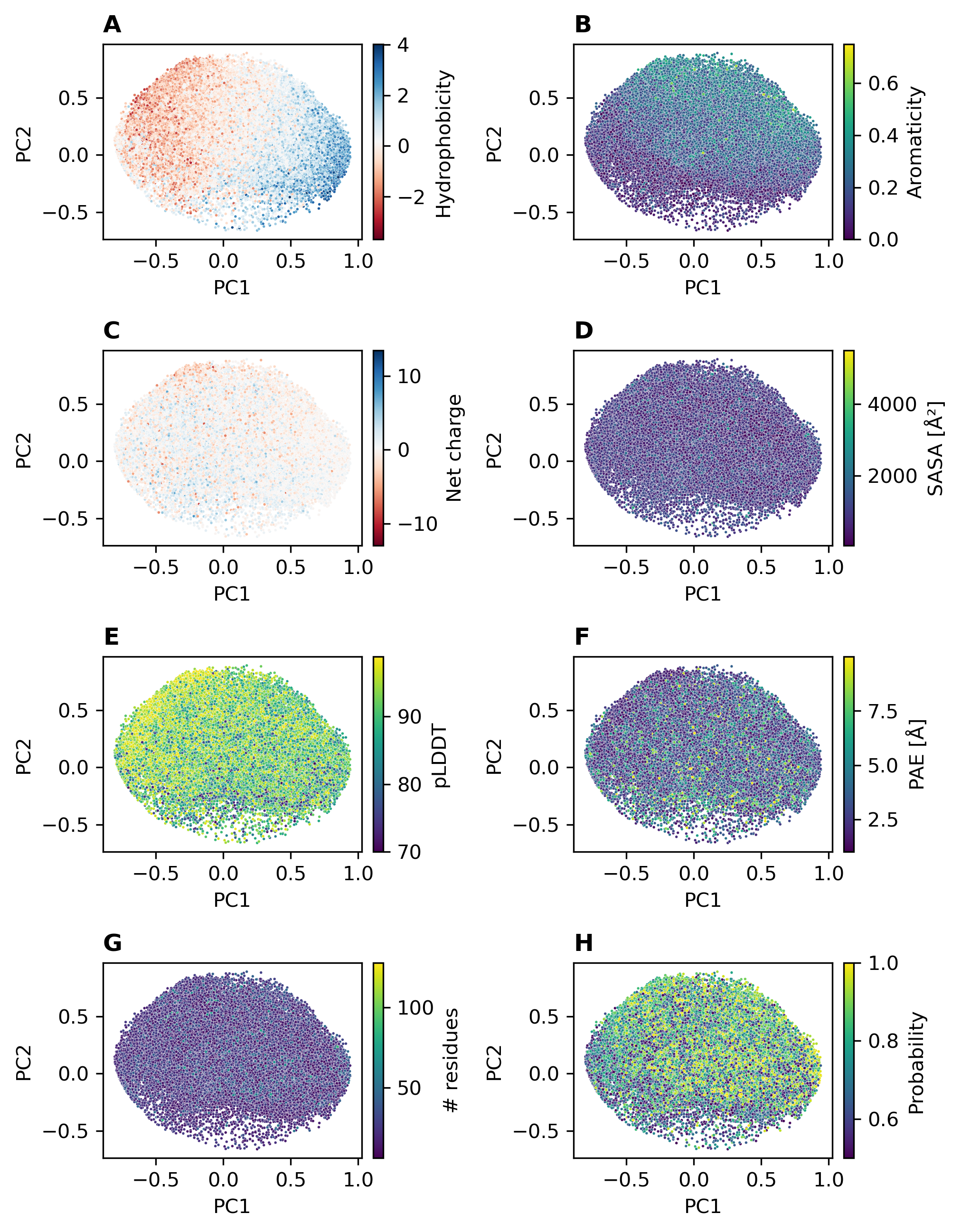

Supplement: S12 Fig — (TIFF) [file pcbi.1013298.s012.tiff]

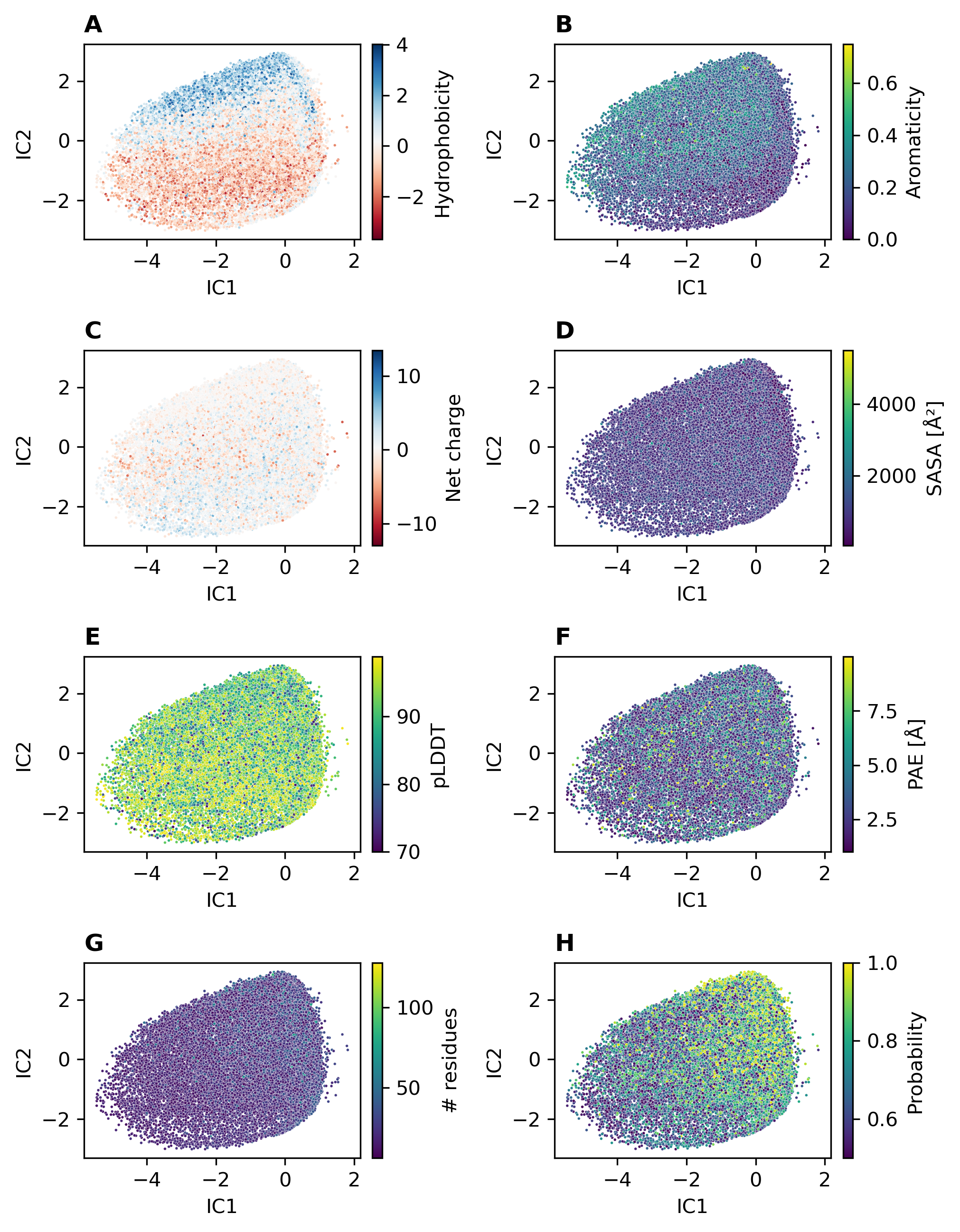

Supplement: S13 Fig — (TIFF) [file pcbi.1013298.s013.tiff]

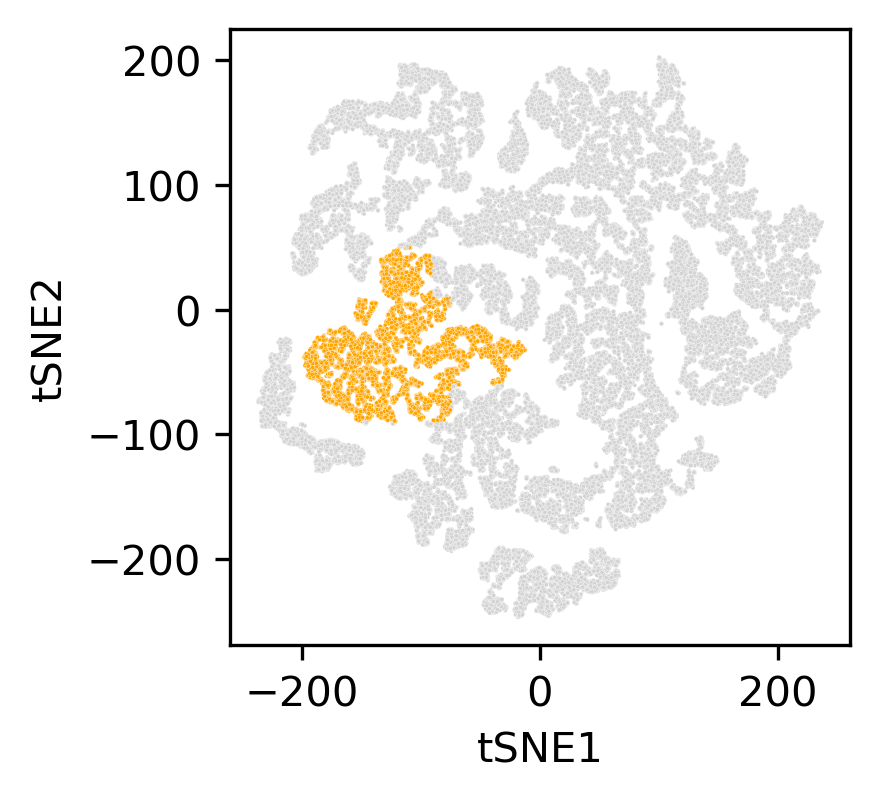

Supplement: S14 Fig — Pockets that are found in the first low probability region are highlighted in orange. All other pockets are colored in orange (TIFF) [file pcbi.1013298.s014.tiff]

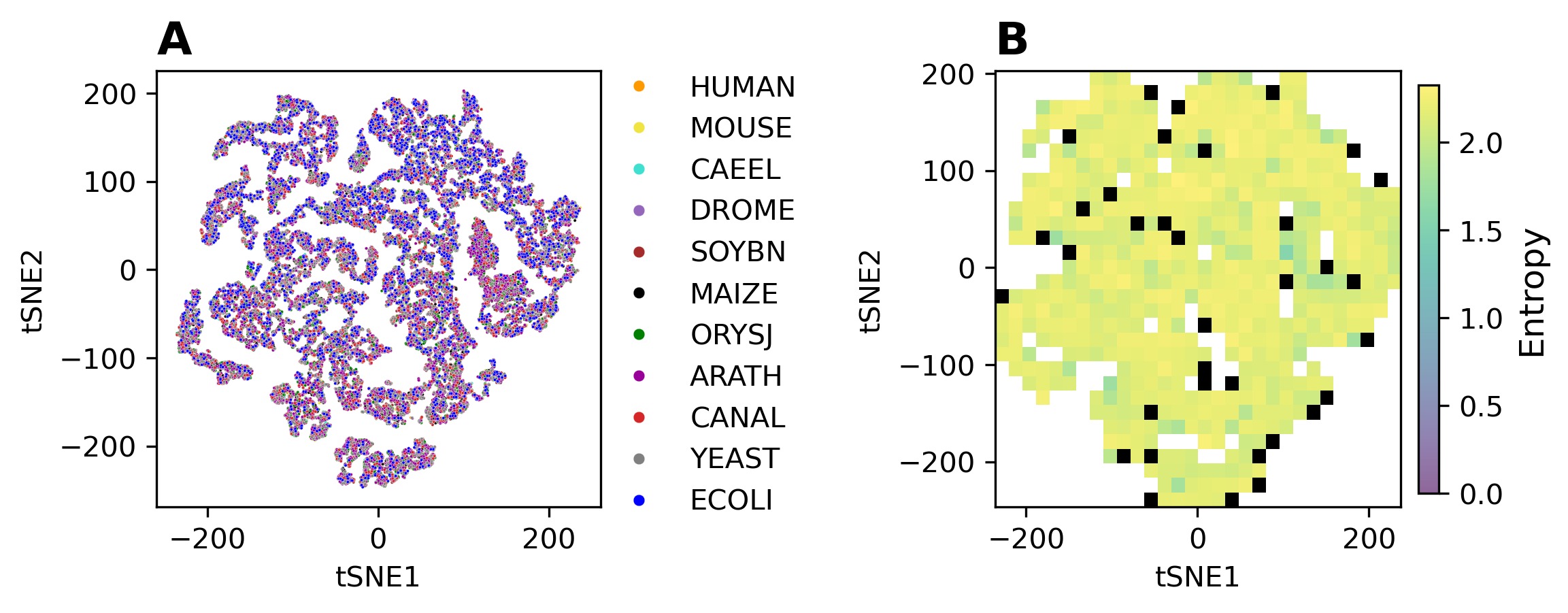

Supplement: S15 Fig — (A) tSNE projection colored by different species. All pockets in their respective tSNE embedding space, (B) Binned entropy, S (see Materials and Methods), heatmap plot, capturing for every square bin the species diversity with low entropy, S, values indicating low diversity and high values corresponding to increased diversity. All grid cells containing fewer than 20 pockets are colored black. (TIFF) [file pcbi.1013298.s015.tiff]
