# Supplementary material for: Towards a comprehensive view of the pocketome universe—biological implications and algorithmic challenges
Source: PLoS Comput Biol. 2025 Jul 24;21(7):e1013298. doi: 10.1371/journal.pcbi.1013298 (PMC12324681; doi:10.1371/journal.pcbi.1013298)
Supplement: Attachment — Submitted filename: Response_letter_pocketome.pdf [file pcbi.1013298.s019.pdf]

PCOMPBIOL-D-25-00258
Towards a comprehensive view of the pocketome universe – biological implications and algorithmic challenges.
PLOS Computational Biology

Dear editor, dear reviewers,

We thank all three reviewers for their time and effort reviewing our study and manuscript. We greatly appreciate the provided constructive critique and have tried to adequately address it to the best of our ability.

Prompted by a very valid comment on our approach with regard to treating overlapping pockets (which we now consider individually rather than going with the pocket of highest confidence only), this revision necessitated a complete re-run of all analysis steps. All previously reported results remained either qualitatively identical or have become more consistent, particularly regarding the reported scaling law (with/without singletons). Naturally, actual numbers had to be updated throughout.

We also added random-control simulations to provide a null-model to which the actual observations (degree-preserved random networks), in particular pertaining to the reported scaling law, can be compared. As a result, we can report more confidently on the sub-linear scaling law, which, as correctly stated by reviewer 3, is now put into the context of the reported/ surmised functional saturation. We feel that with our study, we provide further evidence for this phenomenon, which is reported here for the first time, for pocketome-diversity as a proxy for ligand-binding/processing capacity.

Aside from minor wording improvements, we also made some larger changes with regard to result presentations (instead of UMAP, we now show tSNE embeddings in the main manuscript; we dropped MMseq2 to find and show cluster representatives only, and rather go with all pockets).

Along with addressing all specific points (detailed below), we also expanded on the provided supplementary material that now includes comprehensive pocket information and software code (github).

We also thank the editor for her time and for organizing a timely and high-quality review.

We feel confident that our study and manuscript have greatly benefited from the review and revision process. We hope it can now be endorsed for publication in PLoS Computational Biology.

Hanne Zillmer & Dirk Wather
Golm, Jun 16, 2025

Reviewers' comments:

Reviewer's Responses to Questions

Reviewer #1: This is an important and interesting monumental study of protein binding sites that relies on the library of AlphaFold structures to identify binding sites for a large number of diverse ligands and goes further to compare the characteristics of the sets of binding sites across the broad classes of organisms. The result is an impressive catalog of binding sites of various types across a broad range of organisms, which presents results important for evolutionary information about how binding sites have evolved. Overall, the study is extremely impressive in its breadth and reliance on many different methods.

We appreciate the positive appraisal of our work.

The discussion at the end includes some useful general discussions about factors that could be problematic but lacks some more specific details. Some quantitative testing of the sensitivity of the cutoffs that have been used would be helpful to the reader. The cutoff value of 0.5 used on P2Rank values needs some justification or evaluation of how results change with different cutoff values. Likewise, there is some further information needed regarding the cutoff red line in Figure 1.

We agree. Thresholds need to be set prudently and tested.

Following up on this comment, we assessed the recall of known pockets across different thresholds.

With regard to recall, here is the corresponding statistic:

P2Rank threshold	Found known pockets	not found	ratio found/ all known (recall)2	number of predicted pockets
0	16523	5848	0.739	715522
0.1	14211	8160	0.635	265067
0.2	13250	9121	0.592	176156
0.3	12634	9737	0.565	137531
0.4	12158	10213	0.543	114730
0.5	11662	10709	0.521	97515
0.6	11055	11316	0.494	81068
0.7	10128	12243	0.453	66346
0.8	8529	13842	0.381	50381
0.9	5422	16949	0.242	29361

As can be noted, the percentage recall is quite stable from P2Rank thresholds 0.2 and 0.7. Going lower than 0.5 would result in too many pockets, while going higher in too few (higher false-negative rate).

With regard to the P2Rank confidence value and any othe threshold values, a certain degree of pragmatism has to be applied to false negatives (threshold too high) and false positives (threshold too low). As detailed below, we interrogated our findings with regard to threshold setting much more rigorously in this revision than before (clustering settings etc.))

While actual numbers may differ depending on the threshold settings, qualitatively, no significant differences were observed.

To acknowledge this important comment, we added: “Testing various thresholds, we determined the recall, i.e. the detection of known binding sites quite stable across the P2Rank score threshold range from 0.2-0.7, with 59% and 45%, respectively. To balance the number of true positive and potential false positive binding sites i.e. sites that are not actually involved in any ligand binding, and to arrive at manageable set size (176,156 pockets for threshold of 0.2), a threshold of 0.5 was applied to the probability score as predicted by P2Rank for all pockets. This resulted in a set of 97,515 potential binding sites.

The predicted sets of protein structures from AlphaFold have proven to be extraordinarily informative and are now being used for many different purposes. There are competitive predicted protein structures from other researchers such as RosettaFold and ESMFold but the limited number of comparisons have generally shown that the AlphaFold structures are better. There are also sets of experimentally determined structures that could have been included, but these are woefully incomplete.

One major problem with the AlphaFold protein structures is that many of the structures have long external unfolded segments or segments with only secondary structures, which many researchers are interpreting to be disordered parts or membrane anchors. In principle, these could be affecting some binding sites, but at present I don’t think there is anything reliable or any systematic way to treat these pieces. We and others are simply cutting them off.

We very much relate to this comment in support of our approach (AF with applied quality thresholds). Our applied pLDDT and PAE threshold on the binding pocket lining residues have effectively filtered out such segments/ binding sites.

>In Figure 2, the label derivates should be derivatives.

Corrected.

In the discussion regarding domains, I believe the word domain-no-domain should be domain-to-domain. Also, since the most common motions in proteins are between domains, these cases may be the most affected by the dynamics of the structures.

Our wording was ambiguous on this aspect. We rephrased: “ domain - no_domain interface, i.e. sites for which some residues belong to an annotated domain, while others carry no domain annotation information.”

Reviewer #2: The presented manuscript addresses an intriguing question: whether binding pockets differ across species and how genome size correlates with the number of predicted pockets. To investigate this, the authors analyze AlphaFold-predicted proteomes from several species, apply the P2Rank pocket prediction tool to identify potential binding sites, and use two different similarity assessment methods to evaluate pocket variability both within and between species.

Overall, the manuscript is relatively easy to follow, though some sentences are overly complex, which occasionally hinders comprehension (see comments below). I find the idea of exploring interspecies pocket variation highly interesting, and the scope of the work is solid. However, I have several methodological concerns, outlined below.

We very much appreciate the positive general comments on our study.

Major comments:

-I do not fully understand the rationale for removing proteins with fewer than 100 amino acids. According to [1], about 20% of human proteins are shorter than 100 amino acids (see Fig. 3c). Even if proteins with ion-only binding sites are excluded, this still leaves a substantial number of proteins, likely around 5% (though it is difficult to read from the graph). This exclusion seems arbitrary and may result in a loss of valuable data.

As was noted in Utgés, Javier S., and Geoffrey J. Barton. "Comparative evaluation of methods for the prediction of protein–ligand binding sites." Journal of Cheminformatics 16.1 (2024): 126:

"PUResNet, DeepPocket, $P2Rank_{CONS}$ and P2Rank **often don't predict on smaller proteins (< 100 amino acids)** as well as non-globular or elongated proteins, representing 60–80% of proteins with no predicted pockets".

Because some species, such as ORYSJ, have relatively many short proteins (ORFs), for which many no-pocket proteins would be predicted, which would result in a bias in the statistic.

Therefore, we excluded proteins with fewer than 100 amino acids.

Correspondingly, we added "..., P2Rank has been reported to underpredict on proteins shorter than 100 amino acid residues [ref].

- Pg 6: "If two pockets overlapped, only the pocket that was predicted with a higher probability was kept." -It seems that the situation is not quite right, especially considering that P2Rank predicted pockets, as stated by the authors, are larger than the actual pockets. This implies that two pockets overlapping by a single residue might actually be two distinct pockets. Focusing on only one of them could result in the loss of many valid predictions (though it's unclear how often this happens). Additionally, if we examine Figure 5D from reference [1], we can see that there are indeed several binding sites in the human proteome where residues overlap. In general, the pockets can be quite close to each other, as demonstrated in Figure 5C..

We agree. Our original methodology regarding overlapping pockets was not optimal. In the revision, we have now kept all pockets, irrespective of overlapping residues, as long as they met our other filtering

criteria (pLDDT>70, PAE<10A). As a consequence, a complete new re-run of all analyses was necessary, and all numbers had to be updated. Despite this having been a significant effort, we were pleased with the new sets of results, which proved to be more coherent than before.

It was difficult for me to decode the sentence capturing the main conclusion: "Thus, larger proteomes harbor less than proportionally more different binding sites than species with smaller proteomes." I recommend rephrasing this.

We reworded: “Thus, as proteome size increased during evolution and therefore potentially diversified, the number of distinct binding sites, reflecting potentially diversifying function, grew less than proportionally.”

- The manuscript repeatedly mentions that P2Rank's predicted pockets are much larger than the observed pockets, which improves performance, as reported in [1]. Why wasn’t this mode of prediction used in this study?

We assume that the reviewer refers to the conservation mode, which we have used and now state it explicitly (“... using P2Rank in conservation mode [6]”)

- Pg 14: P2Rank, like many other pocket prediction tools, was not trained on ion-binding data, which limits its performance in this area. This point deserves more discussion.

We would like to draw this reviewer’s attention to our already contained discussion on this point, which we feel is adequate. “The binding sites of inorganic ions are often formed by a few amino acid residues, via charge-driven complex formation or covalently, thereby exhibiting less of the hallmark features of compound binding sites (surface invaginations). This makes it difficult to predict their location if they are bound to a site that is distant from any other binding site. Thus, inorganic ions are proportionally overrepresented in the “not found” set.”

- Tab 4 - I believe both t-SNE and UMAP will produce different embeddings when embedding the data in one dimension versus two dimensions. Therefore, it doesn't make sense to use tSNE1 and tSNE2 independently. If the goal is to correlate t-SNE with a specific property, the data should be projected into one dimension, which would yield different results than projections into two dimensions and the project onto individual axes.

We agree that because tSNE and UMAP are non-linear projection methods that focus on preserving neighbor relationships, in which the embedding axes (dimensions) cannot directly be considered orthogonal and/or independent.

In the revision, we decided to remove this quantitative analysis altogether. Instead, we present a qualitative discussion and have now included all eight tested properties in the main manuscript. (Only four were presented in the main text in the first version, and another four in the supplement. We reconsidered and decided to show them all in the main text, as they all are, in fact, informative.)

With regard to skipping the quantitative analysis. Correlation coefficients (Pearson/Spearman) capture only linearly separable/correlated properties. This is often not the case, in particular in tSNE and UMAP, where the focus is on local neighborhoods and no actual distance metric is used (in the sense of Euclidean/ linear distance).

We considered alternative metrics to assess the uniformity of data distributions, but realized that this is harder than we first thought. In fact, we even believe there is no satisfying solution here, as a) the properties follow different distributions (normal/ bimodal, non-normal, bounded, unbounded), and b) some mean values may compute as (near) zero, e.g. charge, such that standard metrics such as coefficient of variation cannot be applied. Any alternative metric or data transformation seemed unsatisfactory as well.

Therefore, we now present a qualitative discussion, which, we believe, adequately conveys the essence of the projection maps.

- Replicability: I believe the results are not replicable because the authors only provide the UMAP embedding and fail to include the data, predictions, or code for analysis.

We have now enlarged the supplementary material and provide the relevant developed software code in a github repository (for details, please see below).

Minor comments:

- MMseqs2 was used with default settings. What is the default similarity threshold for the version of MMseqs2 used in this study? This should be explicitly stated to provide clarity on the data.

This point is moot as we have decided to go with all pockets rather than representatives, and hence no longer use MMseq2.

-Pg 6: "pockets that include between 4 and 31 residues were considered further" - This seems rather arbitrary. Why was this specific threshold chosen?

In the revision, we have lifted the upper limit altogether and considered all pockets as predicted by P2RANK. On the lower limit, four residues is the actual lower limit of P2RANK.

- Pg8, ln173 - Again, the choice of perplexity for t-SNE seems arbitrary. It is well known that the choice of perplexity significantly impacts clustering. How sensitive are the results to variations in this parameter?

Indeed, setting the perplexity parameter is, given the nature of the tSNE algorithm, arbitrary. We have inspected a range of different values (along with making them available as supplementary material) and considered setting it to 50 a sensible choice. Please note that these plots primarily serve illustrative purposes. No “hard” quantitative results that would depend on the chosen perplexity are being reported.

- The authors mention that ProBis was not used for inter-species comparison due to computational resource limitations. Would it be feasible to run the analysis on high-performance computing (HPC) systems? If so, the resource requirements should be quantified. The manuscript does not provide enough information on how resource-intensive ProBis is.

Indeed, we would have loved to perform a detailed, sensitive all-against-all analysis, but even state-of-the-art, high-performance computing infrastructure available at our institute (several compute servers with up to 256 cores) and beyond (Max Planck Society supercomputing center), an all-against-all

ProBiS comparison proved too long. Already performing the analysis with species was challenging for the species with large proteomes.

- Related to previous - The authors suggest that DeeplyTough is inferior to ProBis. This claim should be supported by a relevant reference.

Here, we report our own experience, and we stand by the noted shortcomings of DeeplyTough in the Discussion, which should be a legitimate approach. We lessened the importance of the reported critiquet by removing it from the Conclusions.

- Pg 8 - I was unfamiliar with this term, so I looked it up. It appears that steerable CNNs are equivariant neural networks (i.e., invariant to rotation). However, I do not understand why data augmentation is needed to achieve rotational invariance, as mentioned in line 161.

Please note that standard CNNs are not rotationally equivariant. They are only equivariant with regard to translation. Evidently, pockets will be presented in various rotations relative to a global reference fram. Thus, standard CNNs would not be appropriate for the task of rotation-invariant embeddings. However, there are approaches to render them rotationally-equirvariant, e.g. streerable filters, the principle behind the method we used. We also tested for rotational-equivariance and confirmed the applied CNN to be indeed insensitive to rotations of the input.

- Pg 9 "The PAE values for each protein of interest were obtained from the AF database, and the pLDDT values were extracted from the corresponding protein structures" - pLDDT also come from the AF database. Sure, they are stored in the PDB files, but they are in the AF database nevertheless.

We rephrased: “The PAE and pLDDT values for each protein of interest were obtained from the AF database, with the pLDDT values extracted directly from the corresponding protein structure files.”

- Pg 9: "were calculated for all obtained physicochemical and AF-quality pocket descriptors correlated with each dimension of the embedding vector, performed for all dimensionality reduction methods." - That reads strange.

In the revision, we decided to remove this quantitative analysis altogether. Please see above for our reasoning (in response to your comment on using the 2D projection dimensions independently)..

- Pg 9: "As DeeplyTough’s embedding space for all binding sites was too densely populated to obtain a meaningful clustering" - I don't think "dense" is an issue for clustering, uniform distribution of the data (which used to be the case when increasing the dimension of data) would be…

Indeed, our wording was not precise. The reviewer is correct. We replaces “densely” by “uniformly” throughout.

-Pg 10: "which has about the same number of predicted pockets as ARATH although it has about 6,000 (~24%) more predicted protein structures than ARATH" - Is this after filtering out small structures?

We have removed the detailed discussion of the subtle differences. Overall, there is a strong correlation between number of protein structures and number of predicted pockets (r=0.98). Hence, this point is moot.

-Fig1 - Why showing PAE and not pLDDT?

We now show both (Fig. 1).

- Fig.1. - Why are the PAE values squared considering they are positive?

The "square" was in reference to the PAE matrix being a square matrix, and not in relation to taking the square of the PAE values. To avoid this confusion, we deleted the term altogether, as it is not needed.

- Ln255: "there are no significant differences of the estimated prediction quality" - statistically? If not, it should be possible to do statistical significance testing.

Here, "no significant differences" was more of a qualitative statement of the distributions being sufficiently similar to be all included in the subsequent analyses and that there are no species-specific AF-effects (as perhaps conceivable as the training set (PDB structures) has widely differing numbers of examples of the different species.) Given the large numbers, even slight differences will become statistically significant, which is not the issue here, rather qualitative equivalence/ effect size (magnitude of difference).

In the revision, we removed "significant" and reworded: "Nowithstanding the species-related differences, we considered all AF-predicted protein structure sets suitable for a systematic cross-species comparison."

- Tab. 2: I believe that majority of the caption should be part of the text.

We agree. We moved the technical description to the M&M section.

- Pg. 17: "This implies that singletons of low probability potentially are pockets that are not actually involved in any ligand binding." - As noted on the PrankWeb website, the probabilities of PrankWeb are calculated on existing structures, i.e., how well the raw score correlates with observed binding sites. That could lead to seeing low scores in non-homologous pockets.

To reflect this aspect, we reworded (addition in bold-face):

"Taken together, singletons differ markedly from non-singleton pockets, which may indicate that they are either special because of their respective particular binding modes and ligands **outside the P2Rank training sets and score-tuning tuning approaches**, or that they may be falsely predicted pockets."

- Pg 18, Ln353/354: N_pocket/N_FS - is this aver or mean. If it is average, could it be driven by outliers?

This ratio does not represent the mean or average per protein. It is the total number of pockets per species and the total number of FoldSeek clusters per species, abbreviated N, as is common practice to denote an absolute number of observations.

- Pg 26: It would be fair to say that clustering is driven by the features of the objects. Different embedding methods will likely result in different clustering, as they view the original data differently. Therefore, the clustering plot represents the feature extraction method’s perspective on the data rather than the underlying data itself, unless there is strong evidence that the feature extraction method truly captures the underlying data. This should be discussed in more detail.

We agree. Though, we wish to point out that original manuscript did already contain such a general disclaimer (“However, it needs to be cautioned that this conclusion is based on the approach pursued here with all the caveats pertaining to the used tools and settings (detailed below).”

In the revision, we invested more efforts on putting our findings on more solid, statistical footings. We performed comptuations on various randomized network version. In particular, the comparison with degree-preserved random networks proved very helpful, as we can now report on actual-vs.-null-model statistics, which lends more credence to the reported less than proportional scaling law.

Also, we tested to greater extent various settings (thresholds etc., stated in M&M, respective result files available under our github page) to make sure that our results are robust (see also our response to the comment of reviewer 1 on the P2Rank threshold). We also tested various cluster settings (resolution parameter in the Leiden algorithm, threshold for turning a weighted into an unweighted graph), with the conclusions that our results are, indeed, robust.

Furthermore, by now comprehensively making our code base available, along the the required input, user can perform their own analyses and test the effect of settings.

Typos:

Ln237 - number pockets -> number of pockets
Ln337 - processes -> processed
Ln346 - proteomes -> proteins (?)
Ln367 - low -> law

Corrected. Thanks.

Reviewer #3: This manuscript presents a large-scale analysis of predicted small-molecule binding pockets (the "pocketome") in protein structures from eleven species, using AlphaFold-predicted 3D models. The authors applied a computational pocket detection method across the full proteomes of selected species from different kingdoms of life, with the aim of characterizing pocket diversity and identifying evolutionary patterns. A notable observation is the identification of a sub-linear scaling law relating the number of unique binding pockets to the number of unique protein structures within a species, suggesting that organisms with more diverse proteomes may not necessarily have proportionally more diverse pocketomes.

While the study is technically competent and the topic remains broadly relevant to computational structural biology, the principal concern is the limited novelty and incremental contribution of the findings. The observed scaling law is interesting, but the concept of saturation or sub-linearity in structure-function space is not new and has been described in earlier literature.

While this reviewer feels that our study is incremental (see our view on this below), we note that this reviewer too commends us on the soundness of our study, as well as appreciating its key finding as “notable”.

We thank this reviewer for pushing us to expand the scope of our discussion. Indeed, we should, and now do, discuss our findings in the context of the reported “saturation of sublinearity in structure-function space”. In fact, we believe that our study provides a meaningful contribution to this surmised phenomenon, in that it aims at the compound-binding capacity developed during evolution, while other publication on the topic discussed function more broadly (GO-terms etc.).

Correspondingly, we added to the Discussion (for actual references, please see manuscript):: “How evolutionary increases in molecular repertoire (genome-size, number of genes etc.) relates to increases in functional diversity has been studied before, in particular in recent years, with close-to-comprehensive coverage of sequence-and-structure space, whether there is evidence of a saturation of sequence-structure-function space exploration by evolution (refs). Notably, Nimwegen (ref) reported on scaling laws relating number of genes with the size of various functional categories (number of genes devoted to the respective functions) based on gene-ontology, found for all but one sub-linear scaling laws, with the notable exception of “signal transduction”, for which higher than linear (exponent 1.55-1.90) were reported, in line with our hypothesis stated in the previous paragraph. With regard to structural space and its association with function, it was repeatedly reported that both same-structure-different-function and different-structure-same-function can be observed (ref). Our results suggest that at large scale, across kindgoms, the functional space (with pocket diversity taken as a proxy of functional capabilities) seems more constrained than structure space and that the predominating mode of evolution is not the invention of novel function using the same fold, as this would have resulted in a greater than linear scaling law, but rather that functional novelty requires disproportionally more novel structures. Interestingly, a recent survey of microbial protein structures and exploiting the recent leap in structure information coverage suggested that structure space is already largely saturated (ref), which, in light of our findings, may mean that functional diversity is also saturated, and has been saturated already earlier (in evolution) than the structural space.”

Additionally, the idea that larger proteomes may include more redundancy (or fewer novel binding sites per structure) is intuitively expected and has been touched on in previous proteome- and ligand-binding studies. Moreover, large-scale surveys of binding pockets across species, including those based on

experimental structures, have been published more than a decade ago—for instance, the study titled "A Comprehensive Survey of Small-Molecule Binding Pockets in Proteins" (2012) and others that followed have examined very similar questions, often with greater scope or methodological innovation. While the current manuscript benefits from the structural coverage provided by AlphaFold, the core scientific insights do not significantly advance the state of knowledge or introduce a clearly novel conceptual framework.

We are aware of related seminal prior works, such as the one quoted by this reviewer (also quoted by us) and others. We believe that our study goes beyond those in that a) thanks to AF, the coverage of protein-structure space has hugely increased and can - with several important and serious caveats, which we address - be considered almost complete, b) we specifically aim at a cross-species/kindom analysis of the pocketomes, which is new. For example, plant proteomes have been hugely underrepresented in all prior studies. Plants, in particular, may deserve a special focus due to their complex secondary metabolism, c) our study employs novel methods, such as the machine-learning-based embedding methods or the used neural network architectures (CNNs) that have been developed by the community.

While developing novel algorithmic approaches is worthwhile, here, given the huge body of work on the topic of identifying and comparing binding sites, we feel that a study that applies current state-of-the-art methods is fully justified, as long as interesting biology is revealed, which we believe is the case.

We also believe that "the idea that larger proteomes may include more redundancy (or fewer novel binding sites per structure)" may indeed be a reasonable hypothesis given the prior publications. Still, until one can study entire proteomes (from a structure perspective - and with all the limitations and caveats), it remains an hypothesis. Here, we set out to test this hypothesis in this new era, into which AF and the likes have propelled us. We are sure that this reviewer agrees that the new breadth of structural information, which represents not just a quantitative, but qualitative leap, warrants a new look.

The conclusions drawn in this study are relatively narrow in scope: the scaling relationship and speculation on evolutionary implications are intriguing but not fully developed or deeply explored.

Prompted not the least by this reviewer, we have expanded on the discussion of our findings in the context of the overall evolutionary structure-function diversity relationship. To fully develop and to explore deeply the evolutionary implications is a tall order indeed, as this reviewer will undoubtedly agree. We hope that with our study, we have provided a meaningful contribution to that quest.

Additionally, the dependence on predicted rather than experimentally validated structures may also limit the interpretability of the results, especially when drawing functional or evolutionary conclusions about binding sites.

It is exactly the opportunity that was created by this new era that was started by AlphaFold (and the likes) that structures are now available for entire proteomes (with all the limitations (complexes, structural flexibility etc.). Until now, all such scaling law studies were either based on samples (experimentally determined structures) or sequences (ever since whole genome/transcriptome sequencing was possible), with the risk of those datasets to be biased according to research interest.

PLOS Computational Biology aims to publish work of broad interest and exceptional significance within the computational biology field. While this study may be of value to researchers working specifically on

protein structure prediction or ligand binding site analysis, the overall contribution does not rise to a level of impact or novelty that aligns with the journal mission. The manuscript may be more suitable for a specialized journal focused on structural bioinformatics or protein annotation.

We graciously leave that decision to the editor.

In summary, the manuscript is well-structured and presents a technically sound analysis, but the lack of conceptual innovation and limited advancement beyond prior work reduce its significance for a general computational biology audience.

We appreciate the positive comments on the soundness of our study. As this reviewer will realize, it was a massive (data-processing-wise) and challenging (conceptually/ algorithmically) undertaking.

Naturally, we view the comments on merit differently. To reiterate: we consider the key new quality to be the unparalleled scale at which our analysis is done (reliably predicted protein structures of whole proteomes, 11 species, cross-kindom). And we feel vindicated by the praise obtained from the two other reviewers.

Have the authors made all data and (if applicable) computational code underlying the findings in their manuscript fully available?
The PLOS Data policy requires authors to make all data and code underlying the findings described in their manuscript fully available without restriction, with rare exception (please refer to the Data Availability Statement in the manuscript PDF file). The data and code should be provided as part of the manuscript or its supporting information, or deposited to a public repository. For example, in addition to summary statistics, the data points behind means, medians and variance measures should be available. If there are restrictions on publicly sharing data or code —e.g. participant privacy or use of data from a third party—those must be specified.

Reviewer #1: Yes

Reviewer #2: **No:** The authors only provide the UMAP embedding and fail to include the data, predictions, or code for analysis.

Reviewer #3: None

We have enlarged the set of supplementary material to include comprehensive tabular information on all binding sites (to include species, AF-code, binding site residues, pocket probability, plddt, PAE, all chemical-physical properties, ligand information, transmembrane annotation).

We also uploaded relevant software code (including user-friendly Jupyter notebooks) and, where possible .e.g. pairwise ProBiS score matrices, the associated input files, to our github repository (https://github.com/zillmerhanne/PocketomeUniverse).
